# Mapping body-related research within the experimental landscape of anorexia nervosa: a scoping review

**DOI:** 10.3389/fnins.2025.1662018

**Published:** 2025-09-17

**Authors:** Sofia Gentili, Valentina Meregalli, Gaia Risso, Serena Giovannini, Eugenia Zambon, Michela Bassolino, Andrea Serino, Angela Favaro, Enrico Collantoni

**Affiliations:** ^1^Department of Neurosciences, University of Padua, Padova, Italy; ^2^Institute of Health, School of Health Sciences, HES-SO Valais-Wallis, Sion, Switzerland; ^3^The Sense Innovation & Research Center, Sion, Lausanne, Switzerland; ^4^MySpace Lab and NeuroRehab Research Center, Service of University Neurorehabilitation (SUN), Lausanne University Hospital, Institution of Lavigny and University of Lausanne, Lausanne, Switzerland; ^5^Padua Neuroscience Center, University of Padua, Padova, Italy

**Keywords:** anorexia nervosa, eating disorders, experimental psychology, body, interoception, multisensory integration

## Abstract

**Introduction:**

Anorexia Nervosa (AN) is a complex psychiatric disorder characterized not only by restrictive eating behaviors and fear of weight gain, but also by emotional dysregulation, cognitive rigidity, and a profound disturbance in bodily experience. While bodily disturbance is clinically central, its multifaceted and pre-reflective nature has made it difficult to investigate experimentally. This scoping review aims to map the experimental case-control literature on AN from the past 15 years, with particular attention to how studies on body representation are represented within the broader field of AN research, both in terms of their prevalence and their subdivision into specific thematic domains.

**Methods:**

A systematic search was conducted on PubMed, Scopus, and Web of Science. Studies were included if they involved an experimental task comparing individuals with AN to healthy controls.

**Results:**

The search yielded six hundred and three eligible studies, each classified into one or more thematic domains: cognition, emotion/social cognition, food-related processing, reward, and body representation. Among these, one hundred and sixty four studies addressed body representation and were further categorized into five subdomains: body image, perception of other bodies, body schema, sensory processing, and interoception.

**Discussion:**

While studies on cognition, emotion, reward, and food processing often used standardized paradigms and showed moderate methodological consistency, research on the bodily domain was notably heterogeneous. This reflects both the conceptual complexity of corporeality and the lack of unified frameworks for its empirical investigation. A recent shift toward multisensory and embodiment-based paradigms suggests increasing interest in implicit and integrative models of body representation. By identifying patterns, gaps, and emerging trends, this review underscores the need for greater conceptual clarity and interdisciplinary convergence. Advancing the experimental study of body representation in AN may support more comprehensive models of the disorder and enhance our understanding of bodily experience in psychiatric conditions.

## 1 Introduction

Anorexia Nervosa (AN) is a complex psychiatric disorder defined by two core symptomatic dimensions: severe dietary restriction and an intense fear of gaining weight. Equally central, however, is a profound disturbance in bodily experience, which affects how individuals perceive, inhabit, and relate to their own bodies (American Psychiatric Association, 2013). Beyond these defining features, the symptomatology of AN is notably broad and multifaceted, encompassing emotional dysregulation, interpersonal difficulties, rigid cognitive and behavioral patterns, impaired decision-making and reward processing, and neurocognitive alterations such as set-shifting deficits and weak central coherence (Miles et al., 2020; Tauro et al., 2022; Zipfel et al., 2015). These clinical dimensions interact in dynamic and often non-linear ways, contributing to the persistence, severity, and marked heterogeneity of the disorder (Treasure et al., 2020). Reflecting this complexity, the experimental literature on AN has expanded considerably over the past decades, spanning a wide range of research domains. A variety of paradigms have been employed to investigate the cognitive, emotional, motivational, and reward-related processes potentially involved in the disorder. However, despite its richness and steady growth, this evidence appears to be distributed across diverse experimental traditions, which may differ in their theoretical assumptions, methodological choices, and operational definitions of core constructs (Glashouwer et al., 2020). This heterogeneity raises important questions about the current structure and balance of empirical research in the field. Among the various domains explored within experimental research on AN, one of the most conceptually and methodologically challenging concerns the notion of corporeality. The centrality of body representation in AN has been recognized since the earliest psychopathological and phenomenological formulations of the disorder. In her seminal work, Hilde Bruch described a pivotal feature of AN as a persistent and pervasive distortion of body representation, not merely as a misjudgment of physical appearance, but as a deeper disruption in the lived experience of one's own body in space and in relation to the self and others (Bruch, 2001). This core psychopathological insight has since been expanded and reformulated by phenomenological scholars, who have characterized AN as a disorder of embodiment, i.e., a disruption in the capacity to experience the body as a coherent, integrated part of the self (Fuchs, 2022). From this perspective, individuals with AN do not simply misperceive their bodies at a visual or cognitive level; rather, they endure a more fundamental disconnection from the bodily self, often expressed as a diminished sense of bodily familiarity, continuity, and agency (Bowden, 2012).

Despite its clinical and psychopathological salience, corporeality in AN has long posed significant challenges to experimental investigation. The concept itself encompasses a wide and heterogeneous range of dimensions, including perceptual body image, sensorimotor integration, spatial representation of the body, interoception, proprioception, and multisensory bodily awareness, each of which may involve distinct neurocognitive and affective mechanisms (de Vignemont, 2010). This conceptual breadth complicates both the definition of corporeality as an experimental object and the development of paradigms capable of capturing its nuances (Bassolino and Serino, 2022). Moreover, the inherently subjective and pre-reflective nature of bodily experience resists full accessibility through conventional behavioral or cognitive neuroscience methodologies.

As a result, empirical studies directly targeting body representation in AN appear relatively fragmented, both in terms of conceptual grounding and methodological cohesion. Compared to research focusing on behavioral (e.g., restrictive eating patterns), neurocognitive (e.g., decision-making, executive control), or emotional (e.g., affective reactivity) aspects of the disorder, experimental investigations into body representation often seem less integrated and less prominent within the broader literature.

Against this background, the present scoping review aims to provide a systematic mapping of the experimental literature on AN over the past 15 years. We begin by assessing the relative distribution of studies across key research domains—such as body, cognition, emotion, reward, and food-related processing—to understand how different aspects of AN have been investigated experimentally. Within this broader landscape, we place particular focus on studies exploring body representation.

By body representation, we refer not only to investigations of body image and body schema, but also to studies examining the perceptual and neural foundations that support these representations—such as unisensory and multisensory processing, including interoception, as well as attitudes and responses toward other bodies. Our aim is to examine how this line of research is situated within the field as a whole, in terms of how extensively it has been investigated compared to other psychological and neurocognitive aspects of AN and how it is organized into specific thematic domains, and to describe the methodological approaches used to study body representation in its perceptual, cognitive, and affective facets.

By identifying overarching patterns, gaps, and areas of overlap, this review seeks to clarify whether and how the study of body representation in AN remains conceptually fragmented. Ultimately, our goal is to inform future research directions that promote more integrated and theoretically grounded approaches to understanding the bodily dimension of AN.

## 2 Methods

A literature search of PubMed, Web of Science, and Scopus databases was conducted to identify peer-reviewed studies that investigated AN using experimental tasks and protocols between January 2009 and February 2025. The following query, applied to both titles and abstracts, was used: (((“anorex^*^” OR “restrict^*^ eat^*^” OR “eating disorder^*^”) AND (“cognitive” OR “behavio^*^” OR “task” OR “stimuli” OR “experimental”)) NOT (“review” [Title] OR “review” [Publication Type] OR “review literature as topic” [MeSH Terms])).

A total of thirty five thousand three hundred and ten studies were found across three databases. A total of thirty thousand two hundred and forty one duplicates were found. Of these, nineteen thousand one hundred and twenty one were deleted, ten thousand seven hundred and sixty four were resolved and three hundred and fifty six were not in principle duplicated. A total of sixteen thousand one hundred and eighty nine records were screened by title and abstract by S.G., V.M., E.C, S.G., G.R., E.Z. to evaluate whether they met the following inclusion criteria: (1) the manuscript was written in English; (2) the study included a group of patients with a current or past diagnosis of AN; (3) the study included at least one experimental task involving objective, behavioral, or performance-based measures (i.e., non self-report), yielding quantitative data; (4) the study was a case-control study comparing individuals with AN to a healthy control group; (5) the manuscript was an original empirical study published in peer-reviewed journals, excluding reviews, meta-analyses, commentaries, conferences and theoretical papers.

**Figure 1:**
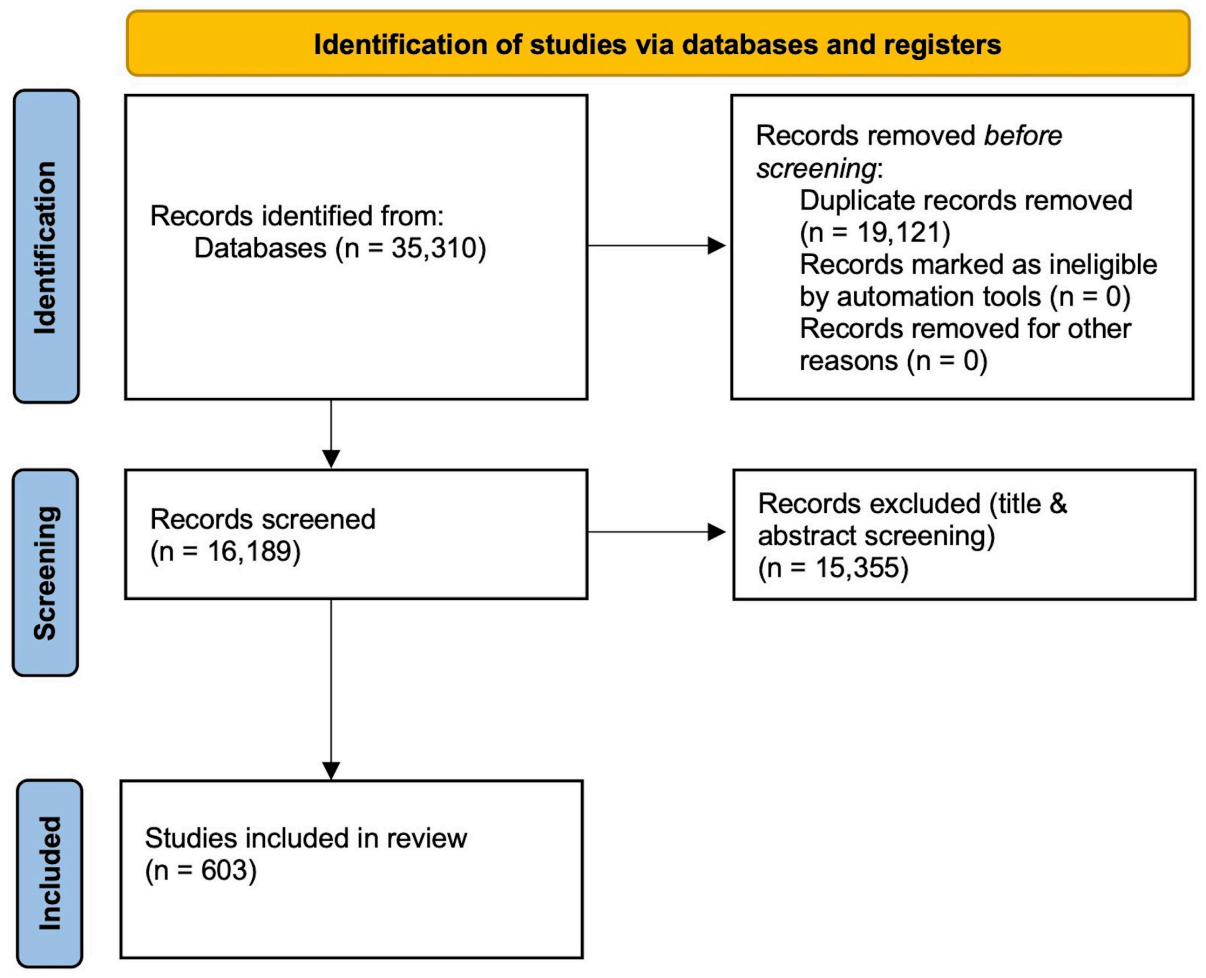
PRISMA flowchart of manuscript selection.

Of these, fifteen thousand five hundred and eighty six articles were excluded according to the above-mentioned criteria and 603 final articles were included (Figure 1). Discussion with E.C. resolved any doubts regarding inclusion.

S.G., V.M. and E.C. double checked the six hundred and three included articles by reading the abstract and assigned labels per macro category to each article. The categories concerned the type of function investigated in each study: cognitive functions (*Cognition*), emotional aspects and social cognition (*Emotion/Social*), processing of food stimuli (*Food*), functioning of the reward system (*Reward*), and body representation (*Body*). Only for studies focusing on the assessment of body representation, sub-categories were created to specify what the task intended to assess. Again, any doubts regarding assignment of the label were resolved by E.C. For each manuscript, the labels and year of publication were extracted.

It should be noted that the screening and category assignment were conducted exclusively by reading titles and abstracts and no full-text assessment of the studies was performed, as the aim was to provide a broad mapping of the literature.

## 3 Results

### 3.1 Main categories

**Figure 2:**
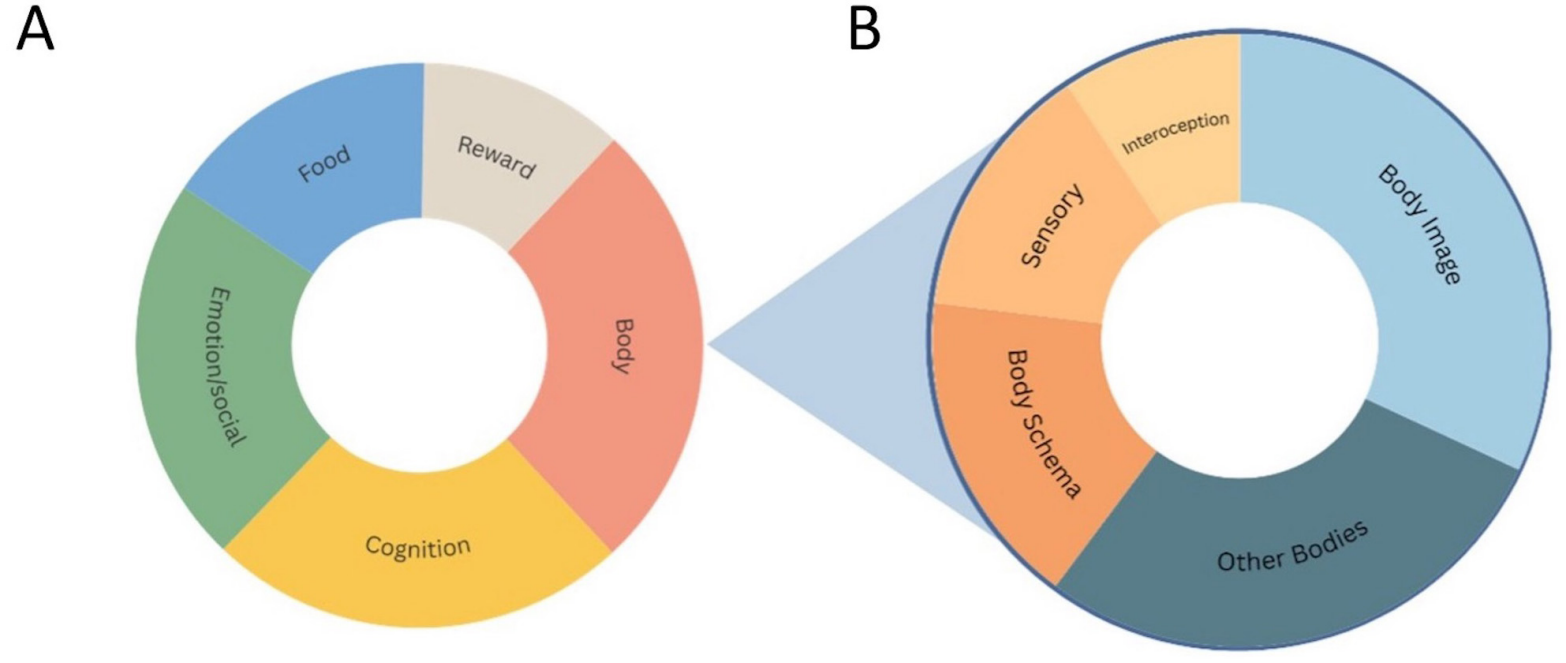
**(A)** Distribution of 603 experimental case-control studies on AN (2009–2025) across five main domains: Cognition, Emotion/Social, Food, Reward, and Body. **(B)** Subdivision of the 164 body-related studies into five subdomains: Body Image, Other Bodies, Body Schema, Sensory Processing, and Interoception.

A total of six hundred and three experimental studies have been conducted in AN over the past 15 years (2009–2025), using a case-control design. Figure 2A illustrates the thematic distribution of these studies. Since some studies addressed more than one domain, they were included in multiple categories; therefore, the sum of studies across thematic areas exceeds the total number of publications. The *Cognition* category (176 studies) comprises studies exploring executive functions, including cognitive flexibility, set-shifting, attentional control, and decision-making. These typically employed computerized neuropsychological tasks to quantify performance on well-established paradigms (e.g., WCST, Stroop tasks, trail-making). The *Emotion/Social* category (163 studies) includes studies investigating emotion recognition, social cognition, and interpersonal functioning. Common tasks involved recognition of facial expressions, eye-tracking during social stimuli presentation, and assessments of emotional awareness and alexithymia. The *Food* (116 studies) category groups studies addressing attentional, emotional, and behavioral responses to food-related cues. These often-used reaction-time paradigms (e.g., dot-probe, Stroop-like tasks), eye-tracking, and neuroimaging to assess approach–avoidance tendencies or food cue reactivity. The *Reward* category (86 studies) explores the functioning of the reward system in AN. It includes studies assessing decision-making and implicit learning processes, sensitivity to reward and punishment, risk evaluation, and preference for immediate vs. delayed rewards. Finally, the *Body* category (164 studies) includes all experimental studies investigating body representation and bodily experience in AN.

### 3.2 Body subcategories

Since the focus of the present study is specifically on experimental research addressing body representation in AN, the *Body* category was further subdivided into thematic areas, presented in Figure 2B. The full list of articles pertaining to the *Body* category, along with their respective subcategories, is provided in the Supplementary material (Supplementary Table 1). Even in this case, since some studies addressed more than one domain, they were included in multiple categories. This classification was developed inductively by the authors based on two key criteria: (1) the main psychological or neurocognitive construct targeted by each study, as inferred from the research aims and methodology; and (2) the underlying conceptual framework employed, particularly with regard to how the notion of body representation was defined or operationalized. In doing so, we aimed to reflect the theoretical diversity of the field while ensuring coherence across empirical paradigms addressing related constructs.

The majority of studies fall within a subcategory focused on the evaluation of *Body Image* (sixty two studies), encompassing perceptual, cognitive, and affective responses to one's own physical appearance. This includes research investigating implicit attitudes and attentional biases toward one's own body image (sixteen studies), studies assessing brain responses to viewing images of one's own body—either photographs or mirror reflections (eight studies), studies on visual self-recognition, self-evaluation, and comparisons with other bodies (eighteen studies, ten of which included neuroimaging), investigations of the effects of exposure to one's own body on mood, eating disorder symptoms, or physiological measures (seven studies), and studies exploring visual perceptual distortions in body size using tasks such as the Figure Rating Scales (thirteen studies).

The second most represented subcategory includes studies investigating how individuals with AN perceive, evaluate, and emotionally react to other people's bodies—typically those of women, and was thus labeled *Other Bodies*. This comprises research on implicit attitudes and attentional processing of other women's bodies (twenty four studies), studies assessing brain responses to viewing images of other women's bodies with different body shapes or during comparisons with these women (twelve studies), investigations of the effects of exposure to other bodies on mood, eating disorder symptoms, or physiological measures (twelve studies), and studies examining size estimation and evaluation of other bodies (six studies).

The third category includes studies addressing the *Body Schema* framework (thirty two studies). This category comprises research investigating the implicit spatial representation of the body, using tasks such as the Door-Like Aperture Task (five studies), studies aiming to modify the implicit body representation through multisensory stimulation, including the Rubber Hand Illusion and Full Body Illusion (nine studies), investigations of the ability of patients with AN to perform cognitive tasks relying on the implicit body schema, such as mental rotation of body parts or visuospatial perspective taking (five studies), studies where patients are asked to implicitly estimate their body size using tasks like tactile distance estimation or localization tasks (nine studies), and studies addressing the representation of the space immediately surrounding the body (four studies).

A fourth subcategory includes studies investigating how individuals with AN perceive and process bodily sensory input, and was therefore labeled *Sensory Processing* (twenty six studies). This category includes studies investigating somatosensory perception, encompassing normal touch, affective touch, and pain perception (eleven studies), studies assessing olfactory functioning (six studies) and visual functioning (four studies), as well as studies addressing multiple sensory modalities and multisensory integration abilities (four studies).

Finally, the last category concerns *Interoception* (eighteen studies). Most of the studies in this category investigate interoceptive accuracy through heartbeat detection tasks (thirteen studies). In addition, some studies examine brain responses during interoceptive attention tasks (two studies), as well as the perception and tolerance of respiratory effort and gastric fullness (two studies).

### 3.3 Temporal trends

**Figure 3:**
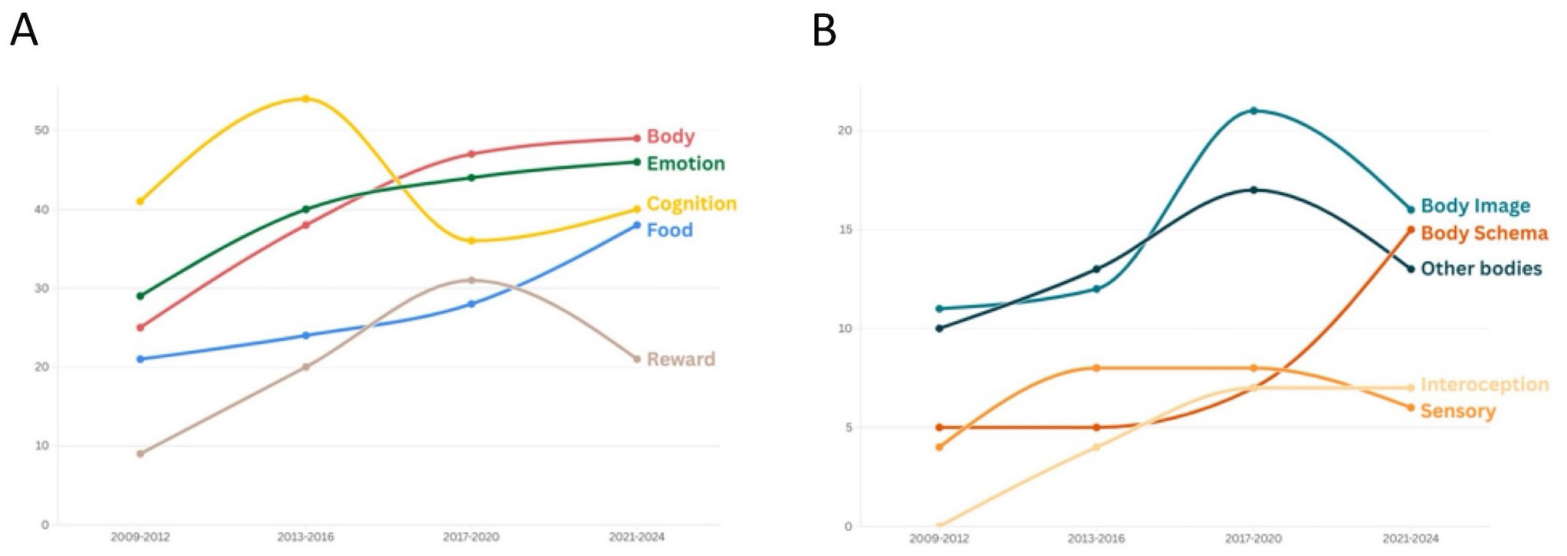
**(A)** Temporal distribution (2009–2024) of experimental case-control studies on AN across the five main research domains. **(B)** Temporal trends within the body-related domain.

Regarding the temporal distribution of the identified macro-domains (Figure 3A), we can observe that during the first period examined (2009–2012), the majority of studies focused on the assessment of cognitive functions. This line of research peaked around 2013–2016, followed by a decline in interest. In fact, in recent years (2020–2024), more studies have focused on body-related processes and emotion/social cognition than on purely cognitive domains. As for more illness-specific aspects, such as the processing of food stimuli and body representation, a steady increase in research interest has been observed over time.

Focusing on the different domains within the body research literature (Figure 3B), we can see that until 2020, most studies addressed body image and the processing of other women's bodies. These research lines peaked between 2017 and 2020 but declined over the past four years. In contrast, there has been growing interest in implicit and multisensory representations of the body, with a substantial increase in studies investigating body schema and interoception.

## 4 Discussion

The present review highlights the breadth and diversity of experimental paradigms applied to the study of AN over the past two decades. Overall, the literature appears relatively balanced across core domains traditionally associated with eating disorders, namely food-related processing and body representation, and other functional domains that are increasingly acknowledged as integral to the multifaceted psychopathology of AN. These include reward functioning, emotional and social processes, and cognitive dimensions such as decision-making, cognitive rigidity, and attentional selectivity. This distribution reflects a growing appreciation for the multidimensional nature of AN and the need to investigate how alterations in affective, social, and executive processes contribute to the emergence and maintenance of core symptoms (Solmi et al., 2018). Within this broader landscape, certain domains, such as food-related processing, stand out for their relatively high degree of methodological and conceptual coherence. In these studies, paradigms typically rely on reaction time measures and attentional indices in response to visually presented food cues, aiming to capture both automatic and controlled responses to disorder-relevant stimuli (Lloyd and Steinglass, 2018). Tasks such as the dot-probe, visual probe, or stroop-like interference paradigms have been widely employed to investigate implicit attentional biases, particularly in relation to approach–avoidance dynamics. While a smaller subset of studies incorporates more overt behavioral outcomes such as actual food choices or consumption, these remain the exception rather than the rule (Foerde et al., 2018). Similarly, emotion-processing studies in AN exhibit a moderate degree of conceptual and methodological cohesion, with most paradigms focusing on facial emotion recognition, gaze-based attention to affective cues, and emotional awareness. Common tasks include standardized facial expression recognition tests (e.g., Ekman faces, “Reading the Mind in the Eyes”), eye-tracking paradigms, and alexithymia assessments (Preti et al., 2022). While primarily centered on explicit behavioral responses such as accuracy and reaction times, some studies also incorporate mood induction or interpersonal contexts, expanding the scope toward social cognition (Monteleone et al., 2020). Despite some variability in design, these studies consistently align with theoretical models emphasizing emotional dysregulation and reduced emotional insight in AN. Studies examining cognitive functioning and reward processing in AN tend to show a moderate degree of methodological consistency, often relying on computerized paradigms derived from established neuropsychological tasks. Frequently used protocols include the Iowa Gambling Task, delay discounting, and monetary reward learning paradigms, which assess decision-making under uncertainty, impulsivity, and sensitivity to reward and punishment (Brown et al., 2024; Tenconi et al., 2016). Other studies investigate cognitive flexibility and executive control using tasks such as the Wisconsin Card Sorting Test (WCST), Stroop tasks, or dedicated set-shifting protocols, which are especially relevant to the cognitive rigidity and weak central coherence commonly described in AN (Tenconi et al., 2010). These paradigms typically provide quantitative and replicable performance indices (such as error rates or reaction times) that support cross-study comparability.

When turning to studies investigating the dimension of corporeality, heterogeneity increases substantially, reflecting the intrinsic complexity and multifaceted nature of this domain. This heterogeneity manifests on two interconnected levels, conceptual and methodological. At the conceptual level, the notion of corporeality encompasses a broad and partly overlapping range of constructs, including body image, body schema, self–other bodily representation, interoception, and multisensory integration. These constructs are rooted in diverse theoretical traditions, ranging from phenomenology and psychopathology to cognitive neuroscience and embodiment theory, and are frequently operationalized without a unified or shared framework (Gadsby, 2017). As a result, experimental studies often approach body-related phenomena from fundamentally different epistemological perspectives, investigating, for instance, perceptual, cognitive, and affective responses to own and others' physical appearance, misperception of body size, disruptions in sensorimotor awareness, or impairments in interoceptive accuracy. This conceptual fragmentation reflects the complexity of bodily experience in AN, which cannot be reduced to a single perceptual distortion but involves deep disruptions in the integration of sensory, motor, and self-related emotional/attitudinal information (Gadsby, 2024). At the methodological level, this conceptual diversity is mirrored by the proliferation of non-standardized experimental protocols, many of which are developed and validated within specific research groups. Paradigms such as the Rubber Hand Illusion (RHI), Full Body Illusion (FBI), mirror exposure tasks, virtual reality-based manipulations, and tactile or interoceptive stimulation procedures differ widely in terms of aims, sensory modality, measurement strategies, procedural design, and output measures (Crucianelli et al., 2021; Engel et al., 2022; Keizer et al., 2014; Provenzano et al., 2024). While these approaches are often innovative and ecologically valid, their variability limits comparability across studies and poses significant challenges for replication. Moreover, the scarcity of normative data and the limited diffusion of standardized tools further hinder the ability to aggregate findings or conduct meta-analytic syntheses. This high degree of heterogeneity stands in stark contrast with the clinical centrality of bodily disturbance in AN. Although the experience of the body is widely acknowledged as a core psychopathological feature, both in classical descriptions and in contemporary models, experimental research in this area remains relatively fragmented and less integrated compared to other domains. This discrepancy may reflect the inherent difficulty of capturing subjective, pre-reflective aspects of body representation using conventional behavioral or neurocognitive methods. It may also signal the need for greater theoretical and methodological convergence, aimed at building cumulative knowledge across studies.

An analysis of publication trends over the past 15 years reveals a clear evolution in the types of research conducted to investigate body representation in AN. Early studies predominantly focused on the assessment of body image representation and perception of one's own and other women's bodies, relying on relatively simple tasks, such as body size estimation task, computerized attentional tasks, and mirror exposure. These types of studies examining body image distortion in AN, consistently show overestimation of body size, negative evaluations of one's own appearance and altered neural responses when viewing one's body. These paradigms, highlighting explicit perceptual and evaluative biases, while foundational, offered limited access to the dynamic, sensorimotor dimensions of corporeal experience, leaving implicit aspects less explored. Over the years, the line of research concerning the perception of other women's bodies, often used as a proxy for social/interpersonal comparison mechanisms in AN, has shifted to a more implicit level. Studies employing implicit association tasks, attentional bias measures, and eye-tracking consistently show heightened attentional capture by thin-ideal bodies and increased negative affect following exposure to others' bodies (Lakritz et al., 2023; Loeber et al., 2016; Meregalli et al., 2025). Neuroimaging evidence further suggests altered activation in reward and salience networks when patients with AN view body of different shapes, underscoring the socio-emotional salience of body-related stimuli in this population (Fladung et al., 2010; Sweitzer et al., 2018). Starting from 2015 and gaining momentum in the years following 2018, research trajectories have increasingly converged toward mechanisms regarding the implicit representations of the body, reflecting an even more pronounced shift in this domain. Paradigms such as the Door-Like Aperture Task, mental rotation of body parts, and perspective-taking tasks reveal difficulties in spatially representing the body and adopting embodied perspectives (Keizer et al., 2013; Lander et al., 2020; Meregalli et al., 2023). Importantly, a noticeable shift also emerges toward multisensory, immersive, and embodiment-oriented paradigms. Techniques such as the RHI, FBI, and virtual reality manipulations become increasingly prevalent. Multisensory paradigms such as RHI and FBI have demonstrated altered susceptibility to manipulations of body ownership in AN, suggesting disturbances in the integration of visual, tactile and proprioceptive information (Keizer et al., 2014; Provenzano et al., 2019, 2024). Furthermore, a smaller but growing literature has examined basic sensory processing in AN, reporting alterations in tactile, affective touch, and pain perception, as well as deficits in olfactory and visual processing (Bär et al., 2013; Davidovic et al., 2018; Dazzi et al., 2013; Li et al., 2015). These findings suggest that even basic sensory channels may be integrated atypically, broadening the scope of corporeal disturbance in AN. Finally, interoceptive processes have received increasing attention, most often assessed via the heartbeat detection task. Other studies extend this evidence to respiratory load and gastric distension, pointing toward a generalized alteration in the processing of internal bodily signal (Berner et al., 2018; Brown et al., 2021; Eshkevari et al., 2014).

Notably, these paradigms allow for the investigation of implicit bodily processes, such as body schema, multisensory integration, and interoception, that had remained largely inaccessible to earlier methodologies. This temporal pattern suggests two complementary developments. On the one hand, it reflects growing technological and computational innovation, which has enabled the implementation of more complex and immersive experimental protocols (Risso and Bassolino, 2022; Serino et al., 2022). On the other hand, it points to a deeper conceptual reframing, from a traditional emphasis on body image as a perceptual and attitudinal judgment to a more integrative understanding of the body as a lived, embodied structure. Nevertheless, despite these advances, the field remains methodologically fragmented. The relative novelty of these paradigms has limited their adoption to a small number of research groups, and their application has largely involved small experimental samples. As such, this branch of the literature remains in a nascent and highly exploratory phase.

In summary, experimental research on AN has explored a range of functional domains, including food-related processing, cognition, emotion, reward and body representation. While this diversity reflects the multifaceted nature of the disorder, the field as a whole still requires more integrated and systematic efforts. In particular, the literature addressing body representation remains notably fragmented, shaped by significant methodological variability and conceptual heterogeneity. This fragmentation reflects, in part, the inherent complexity of corporeality, which spans perceptual, sensorimotor, subjective, and intersubjective dimensions, and is rooted in distinct and often unaligned epistemological frameworks. Recent developments in theoretical and experimental neuroscience, particularly those inspired by embodied and enactive perspectives such as the 4E cognition framework, offer promising avenues to overcome these limitations. However, these approaches remain largely underutilized in the context of AN research. Given the centrality of bodily experience in AN, the disorder may represent a particularly relevant and informative model for advancing embodied theories of mind and self. To support this shift, future research must move toward a more holistic and interdisciplinary vision, combining phenomenological insights, psychopathological models, cognitive neuroscience, and contemporary methodological approaches. Promoting greater theoretical and methodological convergence in this field could improve our understanding of the role of body representation in AN and, more generally, in psychiatric disorders. Due to the centrality of corporeal disturbance in AN, not only in clinical terms, but also at cognitive and experiential levels, this condition may serve as a valuable model for studying how alterations in body representation relate to psychopathology. Experimental research in this area could thus contribute to developing more integrated frameworks for investigating the embodied aspects of mental disorders, ultimately paving the way for novel intervention strategies that specifically target body representation and its disruption in conditions such as AN. The heterogeneity of this literature reflects the multilayered complexity of corporeality in AN and the lack of a unifying explanatory framework. Moving toward more mechanistic accounts will be crucial to identify specific treatment targets, which could be translated into modular, personalized strategies to complement standard evidence-based approaches, thereby enhancing their clinical impact within stepped-care models.

## Data Availability

The original contributions presented in the study are included in the article/Supplementary material, further inquiries can be directed to the corresponding author.
