# Supplementary material for: Mapping body-related research within the experimental landscape of anorexia nervosa: a scoping review
Source: Front Neurosci. 2025 Sep 17;19:1662018. doi: 10.3389/fnins.2025.1662018 (PMC12484205; doi:10.3389/fnins.2025.1662018)
Supplement: Supplementary file 1 [file Table_1.docx]

Supplementary Material

**Supplementary Table 1.** Brief description and subcategorization of studies included in the *Body* category.

| **Study** | **Country** | **Subcategory** | |
| --- | --- | --- | --- |
| Ambrosecchia et al. (2023) | Italy | Body Image | |
| Karakuş Aydos et al. (2024) | Turkey | Body Image | |
| Bauer et al. (2017) | Germany | Body Image | |
| Beato-Fernández et al. (2009) | Spain | Body Image | |
| Beato-Fernández et al. (2011) | Spain | Body Image | |
| Blechert et al. (2011) | Germany | Body Image | |
| Brockmeyer et al. (2018) | Germany | Body Image | |
| Cornelissen et al. (2013) | UK | Body Image | |
| Cornelissen et al. (2017) | UK | Body Image | |
| Crino et al. (2019) | Australia | Body Image | |
| Dalhoff et al. (2019) | Germany | Body Image | |
| Demartini et al. (2021b) | Italy | Body Image | |
| Di Lodovico et al. (2022) | France | Body Image | |
| Fisher et al. (2020) | Germany | Body Image | |
| Forghieri et al. (2016) | Italy | Body Image | |
| Fox et al. (2013) | UK | Body Image | |
| Gadsby et al. (2023) | Belgium | Body Image | |
| Gilon Mann et al. (2018) | Israel | Body Image | |
| Gu et al. (2024) | USA | Body Image | |
| Hartmann et al. (2015) | USA | Body Image | |
| Henn et al. (2022) | Germany | Body Image | |
| Hirot et al. (2016) | France | Body Image | |
| Kazén et al. (2019) | Germany | Body Image | |
| Knejzlíková et al. (2021) | Czechia | Body Image | |
| Lavenne-Collot et al. (2025) | France | Body Image | |
| Lukas et al. (2024) | Germany | Body Image | |
| Martínez-García et al. (2020) | Spain | Body Image | |
| McAdams and Krawczyk (2014) | USA | Body Image | |
| Mendoza et al. (2022) | USA | Body Image | |
| Miyake et al. (2010) | Japan | Body Image | |
| Mohr et al. (2010) | Germany | Body Image | |
| Okamoto et al. (2010) | Japan | Body Image | |
| Phillipou et al. (2015) | Australia | Body Image | |
| Rodriguez-Cano et al. (2009) | Spain | Body Image | |
| Romero Frausto et al. (2024) | Germany | Body Image | |
| Susta et al. (2022) | Slovakia | Body Image | |
| Svaldi et al. (2016) | Germany | Body Image | |
| Terhoeven et al. (2023) | Germany | Body Image | |
| Tuschen-Caffier et al. (2015) | Germany | Body Image | |
| Waldman et al. (2013) | UK | Body Image | |
| Xu et al. (2017) | USA | Body Image | |
| Brown et al. (2021) | USA | Body Image | Body Schema |
| Engel and Keizer (2017) | Netherlands | Body Image | Body Schema |
| Porras-Garcia et al. (2020) | Spain | Body Image | Body Schema |
| Bauer et al. (2017a) | Germany | Body Image | Other Bodies |
| Blechert et al. (2010) | Germany | Body Image | Other Bodies |
| Brockmeyer et al. (2020) | Germany | Body Image | Other Bodies |
| Cazzato et al. (2016) | Italy | Body Image | Other Bodies |
| Couton et al. (2022) | France | Body Image | Other Bodies |
| Friederich et al. (2010) | UK | Body Image | Other Bodies |
| Hartmann et al. (2020) | Germany | Body Image | Other Bodies |
| Kodama et al. (2018) | Japan | Body Image | Other Bodies |
| Legenbauer et al. (2020) | Germany | Body Image | Other Bodies |
| McAdams et al. (2016) | England | Body Image | Other Bodies |
| McAdams et al. (2018) | USA | Body Image | Other Bodies |
| Melles and Jansen (2024) | Netherlands | Body Image | Other Bodies |
| Mölbert et al. (2018) | Germany | Body Image | Other Bodies |
| Via et al. (2018) | Spain | Body Image | Other Bodies |
| Vocks et al. (2010) | Germany | Body Image | Other Bodies |
| Voges et al. (2018) | Germany | Body Image | Other Bodies |
| von Wietersheim et al. (2012) | Germany | Body Image | Other Bodies |
| Beckmann et al. (2021) | Germany | Body Schema | |
| Colle et al. (2023) | Italy | Body Schema | |
| Engel et al. (2022a) | Netherlands | Body Schema | |
| Engel et al. (2022b) | Netherlands | Body Schema | |
| Guardia et al. (2010) | France | Body Schema | |
| Guardia et al. (2012) | France | Body Schema | |
| Guardia et al. (2013) | France | Body Schema | |
| Hasenack et al. (2021) | Netherlands | Body Schema | |
| Keizer et al. (2011) | Netherlands | Body Schema | |
| Keizer et al. (2013) | Netherlands | Body Schema | |
| Keizer et al. (2014) | Netherlands | Body Schema | |
| Lander et al. (2020) | Israel | Body Schema | |
| Longo et al. (2024) | Italy | Body Schema | |
| Meneguzzo et al. (2023) | Italy | Body Schema | |
| Meregalli et al. (2023) | Italy | Body Schema | |
| Mergen et al. (2018) | Germany | Body Schema | |
| Metral et al. (2014) | France | Body Schema | |
| Nandrino et al. (2017) | France | Body Schema | |
| Nico et al. (2010) | Italy | Body Schema | |
| Provenzano et al. (2019) | Italy | Body Schema | |
| Provenzano et al. (2024) | Italy | Body Schema | |
| Scarpina et al. (2022) | Italy | Body Schema | |
| Scarpina et al. (2024) | Italy | Body Schema | |
| Tajadura-Jiménez et al. (2022) | UK | Body Schema | |
| Di Lernia et al. (2023) | Italy | Body Schema | Interoception |
| Keizer et al. (2012) | Netherlands | Body Schema | Sensory |
| Spitoni et al. (2015) | Italy | Body Schema | Sensory |
| Berner et al. (2018) | USA | Interoception | |
| Brown et al. (2022) | USA | Interoception | |
| Demartini et al. (2021a) | Italy | Interoception | |
| Eshkevari et al. (2014) | UK | Interoception | |
| Fischer et al. (2016) | Germany | Interoception | |
| Gajperia et al. (2024) | UK | Interoception | |
| Kerr et al. (2016) | USA | Interoception | |
| Kerr et al. (2017) | USA | Interoception | |
| Kinnaird et al. (2020) | UK | Interoception | |
| Lutz et al. (2019) | Luxembourg | Interoception | |
| Paquet et al. (2024) | France | Interoception | |
| Pollatos et al. (2016) | Germany | Interoception | |
| Richard et al. (2019) | Austria | Interoception | |
| Saramandi et al. (2024) | UK | Interoception | |
| Ambrosecchia et al. (2017) | Italy | Interoception | Body Schema |
| Salvato et al. (2024) | Italy | Interoception | Other Bodies |
| Lapidus et al. (2020) | USA | Interoception | Sensory |
| Arbel et al. (2013) | Israel | Other Bodies | |
| Clarke et al. (2016) | France | Other Bodies | |
| Cornelissen et al. (2016) | UK | Other Bodies | |
| Erdur et al. (2017) | Germany | Other Bodies | |
| Fladung et al. (2010) | Germany | Other Bodies | |
| Fusco et al. (2023) | Italy | Other Bodies | |
| George et al. (2011) | UK | Other Bodies | |
| Horndasch et al. (2012) | Germany | Other Bodies | |
| Horndasch et al. (2018) | Germany | Other Bodies | |
| Horndasch et al. (2020) | Germany | Other Bodies | |
| Horndasch et al. (2023) | Germany | Other Bodies | |
| Kim et al. (2014) | Korea | Other Bodies | |
| Kogel et al. (2021) | Germany | Other Bodies | |
| Kollei et al. (2022) | USA | Other Bodies | |
| Lakritz et al. (2023) | France | Other Bodies | |
| Loeber et al. (2016) | Germany | Other Bodies | |
| Mahr et al. (2022) | USA | Other Bodies | |
| Meregalli et al. (2025) | Italy | Other Bodies | |
| Moody et al. (2017) | USA | Other Bodies | |
| Moody et al. (2021) | USA | Other Bodies | |
| O’Hara et al. (2016) | UK | Other Bodies | |
| Phillipou et al. (2016) | Australia | Other Bodies | |
| Pinhas et al. (2014) | Canada | Other Bodies | |
| Pona et al. (2019) | USA | Other Bodies | |
| Pruis et al. (2012) | USA | Other Bodies | |
| Radix et al. (2023) | Germany | Other Bodies | |
| Sfärlea et al. (2023) | Germany | Other Bodies | |
| Smith et al. (2014) | USA | Other Bodies | |
| Smith et al. (2018) | USA | Other Bodies | |
| Spring and Bulik (2014) | USA | Other Bodies | |
| Sweitzer et al. (2018) | USA | Other Bodies | |
| Urgesi et al. (2012) | Italy | Other Bodies | |
| Urgesi et al. (2014) | England | Other Bodies | |
| Watson (2010) | USA | Other Bodies | |
| Yokokura et al. (2019) | Japan | Other Bodies | |
| Cartaud et al. (2024) | France | Other Bodies | Body Schema |
| Bär et al. (2013) | Germany | Sensory | |
| Bellard et al. (2022) | UK | Sensory | |
| Bischoff-Grethe et al. (2018) | USA | Sensory | |
| Chirico et al. (2019) | Italy | Sensory | |
| Crucianelli et al. (2021) | UK | Sensory | |
| Davidovic et al. (2018) | Sweden | Sensory | |
| Dazzi et al. (2013) | Italy | Sensory | |
| Engel et al. (2020) | Netherlands | Sensory | |
| Fernández-Aranda et al. (2016) | Spain | Sensory | |
| Frost-Karlsson et al. (2022) | Sweden | Sensory | |
| Goldzak-Kunik et al. (2012) | Israel | Sensory | |
| Karavia et al. (2022) | Greece | Sensory | |
| Kirkpatrick et al. (2024) | Canada | Sensory | |
| Li et al. (2015a) | USA | Sensory | |
| Li et al. (2015b) | USA | Sensory | |
| Phillipou et al. (2016b) | Australia | Sensory | |
| Phillipou et al. (2020) | Australia | Sensory | |
| Risso et al. (2020) | Italy | Sensory | |
| Schecklmann et al. (2012) | Germany | Sensory | |
| Stein et al. (2012) | Israel | Sensory | |
| Strigo et al. (2013) | USA | Sensory | |
| Tagini et al. (2023) | Italy | Sensory | |
| Tonacci et al. (2019) | Italy | Sensory | |

**References**

Ambrosecchia, M., Ardizzi, M., Russo, E. C., Ditaranto, F., Speciale, M., Vinai, P., et al. (2023). Bodily self-recognition and body size overestimation in restrictive anorexia nervosa: implicit and explicit mechanisms. *Front Psychol* 14. doi: 10.3389/fpsyg.2023.1197319

Ambrosecchia, M., Ardizzi, M., Russo, E., Ditaranto, F., Speciale, M., Vinai, P., et al. (2017). Interoception and Autonomic Correlates during Social Interactions. Implications for Anorexia. *Front Hum Neurosci* 11. doi: 10.3389/fnhum.2017.00219

Arbel, R., Koren, D., Klein, E., and Latzer, Y. (2013). The neurocognitive basis of insight into illness in anorexia nervosa: A pilot metacognitive study. *Psychiatry Res* 209, 604–610. doi: 10.1016/j.psychres.2013.01.009

Bär, K. ‐J., Berger, S., Schwier, C., Wutzler, U., and Beissner, F. (2013). Insular dysfunction and descending pain inhibition in anorexia nervosa. *Acta Psychiatr Scand* 127, 269–278. doi: 10.1111/j.1600-0447.2012.01896.x

Bauer, A., Schneider, S., Waldorf, M., Braks, K., Huber, T. J., Adolph, D., et al. (2017a). Selective Visual Attention Towards Oneself and Associated State Body Satisfaction: an Eye-Tracking Study in Adolescents with Different Types of Eating Disorders. *J Abnorm Child Psychol* 45, 1647–1661. doi: 10.1007/s10802-017-0263-z

Bauer, A., Schneider, S., Waldorf, M., Cordes, M., Huber, T. J., Braks, K., et al. (2017b). Visual processing of one’s own body over the course of time: Evidence for the vigilance‐avoidance theory in adolescents with anorexia nervosa? *International Journal of Eating Disorders* 50, 1205–1213. doi: 10.1002/eat.22771

Beato-Fernández, L., Rodríguez-Cano, T., and García-Vilches, I. (2011). Psychopathological alterations and neuroimaging findings with discriminant value in eating behavior disorders. *Actas Esp Psiquiatr* 39, 203–2010.

Beato-Fernández, L., Rodríguez-Cano, T., García-Vilches, I., García-Vicente, A., Poblete-García, V., Castrejon, A. S., et al. (2009). Changes in regional cerebral blood flow after body image exposure in eating disorders. *Psychiatry Res Neuroimaging* 171, 129–137. doi: 10.1016/j.pscychresns.2008.01.001

Beckmann, N., Baumann, P., Herpertz, S., Trojan, J., and Diers, M. (2021). How the unconscious mind controls body movements: Body schema distortion in anorexia nervosa. *International Journal of Eating Disorders* 54, 578–586. doi: 10.1002/eat.23451

Bellard, A., Trotter, P., McGlone, F., and Cazzato, V. (2022). Vicarious ratings of self vs. other-directed social touch in women with and recovered from Anorexia Nervosa. *Sci Rep* 12, 13429. doi: 10.1038/s41598-022-17523-2

Berner, L. A., Simmons, A. N., Wierenga, C. E., Bischoff-Grethe, A., Paulus, M. P., Bailer, U. F., et al. (2018). Altered interoceptive activation before, during, and after aversive breathing load in women remitted from anorexia nervosa. *Psychol Med* 48, 142–154. doi: 10.1017/S0033291717001635

Bischoff-Grethe, A., Wierenga, C. E., Berner, L. A., Simmons, A. N., Bailer, U., Paulus, M. P., et al. (2018). Neural hypersensitivity to pleasant touch in women remitted from anorexia nervosa. *Transl Psychiatry* 8, 161. doi: 10.1038/s41398-018-0218-3

Blechert, J., Ansorge, U., Beckmann, S., and Tuschen-Caffier, B. (2011). The undue influence of shape and weight on self-evaluation in anorexia nervosa, bulimia nervosa and restrained eaters: a combined ERP and behavioral study. *Psychol Med* 41, 185–194. doi: 10.1017/S0033291710000395

Blechert, J., Ansorge, U., and Tuschen-Caffier, B. (2010). A body-related dot-probe task reveals distinct attentional patterns for bulimia nervosa and anorexia nervosa. *J Abnorm Psychol* 119, 575–585. doi: 10.1037/a0019531

Brockmeyer, T., Anderle, A., Schmidt, H., Febry, S., Wünsch-Leiteritz, W., Leiteritz, A., et al. (2018). Body image related negative interpretation bias in anorexia nervosa. *Behaviour Research and Therapy* 104, 69–73. doi: 10.1016/j.brat.2018.03.003

Brockmeyer, T., Burdenski, K., Anderle, A., Voges, M. M., Vocks, S., Schmidt, H., et al. (2020). Approach and avoidance bias for thin‐ideal and normal‐weight body shapes in anorexia nervosa. *European Eating Disorders Review* 28, 536–550. doi: 10.1002/erv.2744

Brown, T. A., Perry, T. R., Kaye, W. H., and Wierenga, C. E. (2022). Pilot study of a water load test as a measure of gastric interoception in anorexia nervosa. *Eating and Weight Disorders - Studies on Anorexia, Bulimia and Obesity* 27, 2223–2228. doi: 10.1007/s40519-022-01376-9

Brown, T. A., Shott, M. E., and Frank, G. K. W. (2021). Body size overestimation in anorexia nervosa: Contributions of cognitive, affective, tactile and visual information. *Psychiatry Res* 297, 113705. doi: 10.1016/j.psychres.2021.113705

Cartaud, A., Duriez, P., Querenghi, J., Nandrino, J., Gorwood, P., Viltart, O., et al. (2024). Body shape rather than facial emotion of others alters interpersonal distance in patients with anorexia nervosa. *European Eating Disorders Review* 32, 917–929. doi: 10.1002/erv.3098

Cazzato, V., Mian, E., Mele, S., Tognana, G., Todisco, P., and Urgesi, C. (2016). The effects of body exposure on self-body image and esthetic appreciation in anorexia nervosa. *Exp Brain Res* 234, 695–709. doi: 10.1007/s00221-015-4498-z

Chirico, A., Malighetti, C., Serino, S., Cipresso, P., Pedroli, E., Tuena, C., et al. (2019). Towards an advancement of multisensory integration deficits in anorexia nervosa: Exploring temporal discrimination processing of visuo-auditory stimuli. *Annual Review of Cybertherapy and Telemedicine*.

Clarke, J., Ramoz, N., Fladung, A.-K., and Gorwood, P. (2016). Higher reward value of starvation imagery in anorexia nervosa and association with the Val66Met BDNF polymorphism. *Transl Psychiatry* 6, e829–e829. doi: 10.1038/tp.2016.98

Colle, L., Hilviu, D., Boggio, M., Toso, A., Longo, P., Abbate-Daga, G., et al. (2023). Abnormal sense of agency in eating disorders. *Sci Rep* 13, 14176. doi: 10.1038/s41598-023-41345-5

Cornelissen, K. K., Cornelissen, P. L., Hancock, P. J. B., and Tovée, M. J. (2016). Fixation patterns, not clinical diagnosis, predict body size over‐estimation in eating disordered women and healthy controls. *International Journal of Eating Disorders* 49, 507–518. doi: 10.1002/eat.22505

Cornelissen, K. K., McCarty, K., Cornelissen, P. L., and Tovée, M. J. (2017). Body size estimation in women with anorexia nervosa and healthy controls using 3D avatars. *Sci Rep* 7, 15773. doi: 10.1038/s41598-017-15339-z

Cornelissen, P. L., Johns, A., and Tovée, M. J. (2013). Body size over-estimation in women with anorexia nervosa is not qualitatively different from female controls. *Body Image* 10, 103–111. doi: 10.1016/j.bodyim.2012.09.003

Couton, C., Gorwood, P., Pham‐Scottez, A., Poupon, D., and Duriez, P. (2022). Pupil psychosensory reflex in response to own and standardised silhouettes in patients with anorexia nervosa. *European Eating Disorders Review* 30, 135–145. doi: 10.1002/erv.2881

Crino, N., Touyz, S., and Rieger, E. (2019). How eating disordered and non-eating disordered women differ in their use (and effectiveness) of cognitive self-regulation strategies for managing negative experiences. *Eating and Weight Disorders - Studies on Anorexia, Bulimia and Obesity* 24, 897–904. doi: 10.1007/s40519-017-0448-z

Crucianelli, L., Demartini, B., Goeta, D., Nisticò, V., Saramandi, A., Bertelli, S., et al. (2021). The Anticipation and Perception of Affective Touch in Women with and Recovered from Anorexia Nervosa. *Neuroscience* 464, 143–155. doi: 10.1016/j.neuroscience.2020.09.013

Dalhoff, A. W., Romero Frausto, H., Romer, G., and Wessing, I. (2019). Perceptive Body Image Distortion in Adolescent Anorexia Nervosa: Changes After Treatment. *Front Psychiatry* 10. doi: 10.3389/fpsyt.2019.00748

Davidovic, M., Karjalainen, L., Starck, G., Wentz, E., Björnsdotter, M., and Olausson, H. (2018). Abnormal brain processing of gentle touch in anorexia nervosa. *Psychiatry Res Neuroimaging* 281, 53–60. doi: 10.1016/j.pscychresns.2018.08.007

Dazzi, F., Nitto, S. De, Zambetti, G., Loriedo, C., and Ciofalo, A. (2013). Alterations of the Olfactory–Gustatory Functions in Patients with Eating Disorders. *European Eating Disorders Review* 21, 382–385. doi: 10.1002/erv.2238

Demartini, B., Goeta, D., Marchetti, M., Bertelli, S., Anselmetti, S., Cocchi, A., et al. (2021a). The effect of a single yoga class on interoceptive accuracy in patients affected by anorexia nervosa and in healthy controls: a pilot study. *Eating and Weight Disorders - Studies on Anorexia, Bulimia and Obesity* 26, 1427–1435. doi: 10.1007/s40519-020-00950-3

Demartini, B., Nisticò, V., Tedesco, R., Marzorati, A., Ferrucci, R., Priori, A., et al. (2021b). Visual perception and dissociation during Mirror Gazing Test in patients with anorexia nervosa: a preliminary study. *Eating and Weight Disorders - Studies on Anorexia, Bulimia and Obesity* 26, 1541–1551. doi: 10.1007/s40519-020-00977-6

Di Lernia, D., Serino, S., Tuena, C., Cacciatore, C., Polli, N., and Riva, G. (2023). Mental health meets computational neuroscience: A predictive Bayesian account of the relationship between interoception and multisensory bodily illusions in anorexia nervosa. *International Journal of Clinical and Health Psychology* 23, 100383. doi: 10.1016/j.ijchp.2023.100383

Di Lodovico, L., Hanachi, M., Duriez, P., and Gorwood, P. (2022). The Fitter I Am, the Larger I Feel—The Vicious Circle of Physical Exercise in Anorexia Nervosa. *Nutrients* 14, 4507. doi: 10.3390/nu14214507

Engel, M. M., Ainley, V., Tsakiris, M., Chris Dijkerman, H., and Keizer, A. (2022a). Sense of agency during and following recovery from anorexia nervosa. *Conscious Cogn* 103, 103369. doi: 10.1016/j.concog.2022.103369

Engel, M. M., Gadsby, S., Corcoran, A. W., Keizer, A., Dijkerman, H. C., and Hohwy, J. (2022b). Waiting longer, feeling fatter: Effects of response delay on tactile distance estimation and confidence in females with anorexia nervosa. *Brain Behav* 12. doi: 10.1002/brb3.2422

Engel, M. M., and Keizer, A. (2017). Body representation disturbances in visual perception and affordance perception persist in eating disorder patients after completing treatment. *Sci Rep* 7, 16184. doi: 10.1038/s41598-017-16362-w

Engel, M. M., van Denderen, K., Bakker, A.-R., Corcoran, A. W., Keizer, A., and Dijkerman, H. C. (2020). Anorexia nervosa and the size-weight illusion: No evidence of impaired visual-haptic object integration. *PLoS One* 15, e0237421. doi: 10.1371/journal.pone.0237421

Erdur, L., Weber, C., Zimmermann‐Viehoff, F., Rose, M., and Deter, H. (2017). Affective Responses in Different Stages of Anorexia Nervosa: Results from a Startle‐reflex Paradigm. *European Eating Disorders Review* 25, 114–122. doi: 10.1002/erv.2502

Eshkevari, E., Rieger, E., Musiat, P., and Treasure, J. (2014). An Investigation of Interoceptive Sensitivity in Eating Disorders Using a Heartbeat Detection Task and a Self‐report Measure. *European Eating Disorders Review* 22, 383–388. doi: 10.1002/erv.2305

Fernández-Aranda, F., Agüera, Z., Fernández-García, J. C., Garrido-Sanchez, L., Alcaide-Torres, J., Tinahones, F. J., et al. (2016). Smell–taste dysfunctions in extreme weight/eating conditions: analysis of hormonal and psychological interactions. *Endocrine* 51, 256–267. doi: 10.1007/s12020-015-0684-9

Fischer, D., Berberich, G., Zaudig, M., Krauseneck, T., Weiss, S., and Pollatos, O. (2016). Interoceptive Processes in Anorexia Nervosa in the Time Course of Cognitive-Behavioral Therapy: A Pilot Study. *Front Psychiatry* 7. doi: 10.3389/fpsyt.2016.00199

Fisher, S., Abdullah, A., Charvin, I., Da Fonseca, D., and Bat-Pitault, F. (2020). Comparison of body image evaluation by virtual reality and paper-based figure rating scales in adolescents with anorexia nervosa: retrospective study. *Eating and Weight Disorders - Studies on Anorexia, Bulimia and Obesity* 25, 735–743. doi: 10.1007/s40519-019-00680-1

Fladung, A.-K., Grön, G., Grammer, K., Herrnberger, B., Schilly, E., Grasteit, S., et al. (2010). A Neural Signature of Anorexia Nervosa in the Ventral Striatal Reward System. *American Journal of Psychiatry* 167, 206–212. doi: 10.1176/appi.ajp.2009.09010071

Forghieri, M., Monzani, D., Mackinnon, A., Ferrari, S., Gherpelli, C., and Galeazzi, G. M. (2016). Posturographic destabilization in eating disorders in female patients exposed to body image related phobic stimuli. *Neurosci Lett* 629, 155–159. doi: 10.1016/j.neulet.2016.07.002

Fox, J. R. E., Smithson, E., Baillie, S., Ferreira, N., Mayr, I., and Power, M. J. (2013). Emotion Coupling and Regulation in Anorexia Nervosa. *Clin Psychol Psychother* 20, 319–333. doi: 10.1002/cpp.1823

Friederich, H.-C., Brooks, S., Uher, R., Campbell, I. C., Giampietro, V., Brammer, M., et al. (2010). Neural correlates of body dissatisfaction in anorexia nervosa. *Neuropsychologia* 48, 2878–2885. doi: 10.1016/j.neuropsychologia.2010.04.036

Frost-Karlsson, M., Capusan, A. J., Perini, I., Olausson, H., Zetterqvist, M., Gustafsson, P. A., et al. (2022). Neural processing of self-touch and other-touch in anorexia nervosa and autism spectrum condition. *Neuroimage Clin* 36, 103264. doi: 10.1016/j.nicl.2022.103264

Fusco, G., Ciccarone, S., Petrucci, M., Cozzani, B., Vercelli, G., Cotugno, A., et al. (2023). Altered processing of conflicting body representations in women with restrictive anorexia nervosa. *Psychol Res* 87, 1696–1709. doi: 10.1007/s00426-022-01788-3

Gadsby, S., Zopf, R., Brooks, K. R., Schumann, A., de la Cruz, F., Rieger, K., et al. (2023). Testing visual self‐misperception in anorexia nervosa using a symmetrical body size estimation paradigm. *International Journal of Eating Disorders* 56, 2149–2154. doi: 10.1002/eat.24030

Gajperia, C., McBride, J., Treasure, J., Cardi, V., and Brewer, R. (2024). Recognition of others’ interoceptive states in those with and without eating disorders. *BMC Psychiatry* 24, 169. doi: 10.1186/s12888-024-05615-4

George, H. R., Cornelissen, P. L., Hancock, P. J. B., Kiviniemi, V. V., and Tovée, M. J. (2011). Differences in eye-movement patterns between anorexic and control observers when judging body size and attractiveness. *British Journal of Psychology* 102, 340–354. doi: 10.1348/000712610X524291

Gilon Mann, T., Hamdan, S., Bar‐Haim, Y., Lazarov, A., Enoch‐Levy, A., Dubnov‐Raz, G., et al. (2018). Different attention bias patterns in anorexia nervosa restricting and binge/purge types. *European Eating Disorders Review* 26, 293–301. doi: 10.1002/erv.2593

Goldzak-Kunik, G., Friedman, R., Spitz, M., Sandler, L., and Leshem, M. (2012). Intact sensory function in anorexia nervosa. *Am J Clin Nutr* 95, 272–282. doi: 10.3945/ajcn.111.020131

Gu, S. J., Aimufua, I., Pagliaccio, D., Shankman, S. A., Steinglass, J. E., Auerbach, R. P., et al. (2024). Self‐referential processing in anorexia nervosa. *International Journal of Eating Disorders* 57, 1234–1244. doi: 10.1002/eat.24176

Guardia, D., Carey, A., Cottencin, O., Thomas, P., and Luyat, M. (2013). Disruption of Spatial Task Performance in Anorexia Nervosa. *PLoS One* 8, e54928. doi: 10.1371/journal.pone.0054928

Guardia, D., Conversy, L., Jardri, R., Lafargue, G., Thomas, P., Dodin, V., et al. (2012). Imagining One’s Own and Someone Else’s Body Actions: Dissociation in Anorexia Nervosa. *PLoS One* 7, e43241. doi: 10.1371/journal.pone.0043241

Guardia, D., Lafargue, G., Thomas, P., Dodin, V., Cottencin, O., and Luyat, M. (2010). Anticipation of body-scaled action is modified in anorexia nervosa. *Neuropsychologia* 48, 3961–3966. doi: 10.1016/j.neuropsychologia.2010.09.004

Hartmann, A., Borgers, T., Thomas, J. J., Giabbiconi, C., and Vocks, S. (2020). Faced with one’s fear: Attentional bias in anorexia nervosa and healthy individuals upon confrontation with an obese body stimulus in an eye‐tracking paradigm. *Brain Behav* 10. doi: 10.1002/brb3.1834

Hartmann, A. S., Thomas, J. J., Greenberg, J. L., Elliott, C. M., Matheny, N. L., and Wilhelm, S. (2015). Anorexia nervosa and body dysmorphic disorder: A comparison of body image concerns and explicit and implicit attractiveness beliefs. *Body Image* 14, 77–84. doi: 10.1016/j.bodyim.2015.03.013

Hasenack, B., Sternheim, L., Bijsterbosch, J., and Keizer, A. (2021). The link between anxiety and assessment of body attitudes and body size estimation in anorexia nervosa. *J Exp Psychopathol* 12. doi: 10.1177/2043808721997632

Henn, A. T., Borgers, T., Vocks, S., Giabbiconi, C.-M., and Hartmann, A. S. (2022). Visualizing Emotional Arousal within the Context of Body Size Evaluation: A Pilot Study of Steady-State Visual Evoked Potentials in Women with Anorexia Nervosa and Healthy Controls. *Body Image* 40, 78–91. doi: 10.1016/j.bodyim.2021.11.004

Hirot, F., Lesage, M., Pedron, L., Meyer, I., Thomas, P., Cottencin, O., et al. (2016). Impaired processing of self-face recognition in anorexia nervosa. *Eating and Weight Disorders - Studies on Anorexia, Bulimia and Obesity* 21, 31–40. doi: 10.1007/s40519-015-0223-y

Horndasch, S., Heinrich, H., Kratz, O., and Moll, G. H. (2012). The late positive potential as a marker of motivated attention to underweight bodies in girls with anorexia nervosa. *J Psychosom Res* 73, 443–447. doi: 10.1016/j.jpsychores.2012.09.020

Horndasch, S., Kratz, O., Van Doren, J., Graap, H., Kramer, R., Moll, G. H., et al. (2018). Cue reactivity towards bodies in anorexia nervosa – common and differential effects in adolescents and adults. *Psychol Med* 48, 508–518. doi: 10.1017/S0033291717001994

Horndasch, S., Rösch, J., Kratz, O., Vogel, A., Heinrich, H., Graap, H., et al. (2020). Neural mechanisms of perceptive and affective processing of body stimuli in anorexia nervosa – are there developmental effects? *Psychiatry Res* 286, 112853. doi: 10.1016/j.psychres.2020.112853

Horndasch, S., Sharon, E., Eichler, A., Graap, H., Moll, G. H., and Kratz, O. (2023). Heart Rate as a Correlate for the Emotional Processing of Body Stimuli in Anorexia Nervosa. *Behavioral Sciences* 13, 215. doi: 10.3390/bs13030215

Karakuş Aydos, Y., Dövencioğlu, D., Karlı Oğuz, K., Özdemir, P., Pehlivantürk Kızılkan, M., Kanbur, N., et al. (2024). Neural correlates of distorted body images in adolescent girls with anorexia nervosa: How is it different from major depressive disorder? *J Neuropsychol* 18, 154–172. doi: 10.1111/jnp.12340

Karavia, A., Kapsali, F., Gonidakis, F., Koliou, A., Tsigkaropoulou, E., Papageorgiou, C., et al. (2022). Olfactory capacity in anorexia nervosa: correlations with set-shifting ability. *Eating and Weight Disorders - Studies on Anorexia, Bulimia and Obesity* 27, 535–542. doi: 10.1007/s40519-021-01188-3

Kazén, M., Baumann, N., Twenhöfel, J. F., and Kuhl, J. (2019). When do anorexic patients perceive their body as too fat? Aggravating and ameliorating factors. *PLoS One* 14, e0212612. doi: 10.1371/journal.pone.0212612

Keizer, A., Smeets, M. A. M., Dijkerman, H. C., Uzunbajakau, S. A., van Elburg, A., and Postma, A. (2013). Too Fat to Fit through the Door: First Evidence for Disturbed Body-Scaled Action in Anorexia Nervosa during Locomotion. *PLoS One* 8, e64602. doi: 10.1371/journal.pone.0064602

Keizer, A., Smeets, M. A. M., Dijkerman, H. C., van den Hout, M., Klugkist, I., van Elburg, A., et al. (2011). Tactile body image disturbance in anorexia nervosa. *Psychiatry Res* 190, 115–120. doi: 10.1016/j.psychres.2011.04.031

Keizer, A., Smeets, M. A. M., Dijkerman, H. C., van Elburg, A., and Postma, A. (2012). Aberrant somatosensory perception in Anorexia Nervosa. *Psychiatry Res* 200, 530–537. doi: 10.1016/j.psychres.2012.05.001

Keizer, A., Smeets, M. A. M., Postma, A., van Elburg, A., and Dijkerman, H. C. (2014). Does the experience of ownership over a rubber hand change body size perception in anorexia nervosa patients? *Neuropsychologia* 62, 26–37. doi: 10.1016/j.neuropsychologia.2014.07.003

Kerr, K. L., Moseman, S. E., Avery, J. A., Bodurka, J., and Simmons, W. K. (2017). Influence of Visceral Interoceptive Experience on the Brain’s Response to Food Images in Anorexia Nervosa. *Psychosom Med* 79, 777–784. doi: 10.1097/PSY.0000000000000486

Kerr, K. L., Moseman, S. E., Avery, J. A., Bodurka, J., Zucker, N. L., and Simmons, W. K. (2016). Altered Insula Activity during Visceral Interoception in Weight-Restored Patients with Anorexia Nervosa. *Neuropsychopharmacology* 41, 521–528. doi: 10.1038/npp.2015.174

Kim, Y.-R., Kim, C.-H., Cardi, V., Eom, J.-S., Seong, Y., and Treasure, J. (2014). Intranasal oxytocin attenuates attentional bias for eating and fat shape stimuli in patients with anorexia nervosa. *Psychoneuroendocrinology* 44, 133–142. doi: 10.1016/j.psyneuen.2014.02.019

Kinnaird, E., Stewart, C., and Tchanturia, K. (2020). Interoception in Anorexia Nervosa: Exploring Associations With Alexithymia and Autistic Traits. *Front Psychiatry* 11. doi: 10.3389/fpsyt.2020.00064

Kirkpatrick, R. H., Booij, L., Riek, H. C., Huang, J., Pitigoi, I. C., Brien, D. C., et al. (2024). Oculomotor behaviors in youth with an eating disorder: findings from a video-based eye tracking task. *J Eat Disord* 12, 121. doi: 10.1186/s40337-024-01084-y

Knejzlíková, T., Světlák, M., Malatincová, T., Roman, R., Chládek, J., Najmanová, J., et al. (2021). Electrodermal Response to Mirror Exposure in Relation to Subjective Emotional Responses, Emotional Competences and Affectivity in Adolescent Girls With Restrictive Anorexia and Healthy Controls. *Front Psychol* 12. doi: 10.3389/fpsyg.2021.673597

Kodama, N., Moriguchi, Y., Takeda, A., Maeda, M., Ando, T., Kikuchi, H., et al. (2018). Neural correlates of body comparison and weight estimation in weight-recovered anorexia nervosa: a functional magnetic resonance imaging study. *Biopsychosoc Med* 12, 15. doi: 10.1186/s13030-018-0134-z

Kogel, A., Herpertz, S., Steins‐Loeber, S., and Diers, M. (2021). Disorder specific rewarding stimuli in anorexia nervosa. *International Journal of Eating Disorders* 54, 1477–1485. doi: 10.1002/eat.23526

Kollei, I., Leins, J., Rinck, M., Waldorf, M., Kuhn, M., Rauh, E., et al. (2022). Implicit approach‐avoidance tendencies toward food and body stimuli absent in individuals with anorexia nervosa, bulimia nervosa, and healthy controls. *International Journal of Eating Disorders* 55, 85–97. doi: 10.1002/eat.23638

Lakritz, C., Iceta, S., Duriez, P., Makdassi, M., Masetti, V., Davidenko, O., et al. (2023). Measuring implicit associations between food and body stimuli in anorexia nervosa: a Go/No-Go Association Task. *Eating and Weight Disorders - Studies on Anorexia, Bulimia and Obesity* 28, 93. doi: 10.1007/s40519-023-01621-9

Lander, R., Heled, E., and Gur, E. (2020). Executive functioning and spatial processing in anorexia nervosa: an experimental study and its significance for the allocentric lock theory. *Eating and Weight Disorders - Studies on Anorexia, Bulimia and Obesity* 25, 1039–1047. doi: 10.1007/s40519-019-00728-2

Lapidus, R. C., Puhl, M., Kuplicki, R., Stewart, J. L., Paulus, M. P., Rhudy, J. L., et al. (2020). Heightened affective response to perturbation of respiratory but not pain signals in eating, mood, and anxiety disorders. *PLoS One* 15, e0235346. doi: 10.1371/journal.pone.0235346

Lavenne-Collot, N., Maubant, E., Déroulez, S., Bronsard, G., Wehrmann, M., Botbol, M., et al. (2025). Self /other recognition and distinction in adolescents with anorexia nervosa: A pilot study using a double mirror paradigm. *PLoS One* 20, e0309548. doi: 10.1371/journal.pone.0309548

Legenbauer, T., Radix, A. K., Naumann, E., and Blechert, J. (2020). The Body Image Approach Test (BIAT): A Potential Measure of the Behavioral Components of Body Image Disturbance in Anorexia and Bulimia Nervosa? *Front Psychol* 11. doi: 10.3389/fpsyg.2020.00030

Li, W., Lai, T. M., Bohon, C., Loo, S. K., McCurdy, D., Strober, M., et al. (2015a). Anorexia nervosa and body dysmorphic disorder are associated with abnormalities in processing visual information. *Psychol Med* 45, 2111–2122. doi: 10.1017/S0033291715000045

Li, W., Lai, T. M., Loo, S. K., Strober, M., Mohammad-Rezazadeh, I., Khalsa, S., et al. (2015b). Aberrant early visual neural activity and brain-behavior relationships in anorexia nervosa and body dysmorphic disorder. *Front Hum Neurosci* 9. doi: 10.3389/fnhum.2015.00301

Loeber, S., Burgmer, R., Wyssen, A., Leins, J., Rustemeier, M., Munsch, S., et al. (2016). Short‐term effects of media exposure to the thin ideal in female inpatients with an eating disorder compared to female inpatients with a mood or anxiety disorder or women with no psychiatric disorder. *International Journal of Eating Disorders* 49, 708–715. doi: 10.1002/eat.22524

Longo, P., Scaliti, E., Panero, M., Toppino, F., Brustolin, A., Salis, B., et al. (2024). A quantitative study on peripersonal space in anorexia nervosa and healthy subjects: Role of social variables and association with psychopathology. *International Journal of Clinical and Health Psychology* 24, 100476. doi: 10.1016/j.ijchp.2024.100476

Lukas, L., Nuding, L., Schulte‐Körne, G., Platt, B., and Sfärlea, A. (2024). Seeing oneself as an unattractive loser: Similar interpretation and memory biases in adolescents with anorexia nervosa and adolescents with depression or anxiety. *European Eating Disorders Review* 32, 855–868. doi: 10.1002/erv.3095

Lutz, A. P. C., Schulz, A., Voderholzer, U., Koch, S., van Dyck, Z., and Vögele, C. (2019). Enhanced cortical processing of cardio-afferent signals in anorexia nervosa. *Clinical Neurophysiology* 130, 1620–1627. doi: 10.1016/j.clinph.2019.06.009

Mahr, F., Bunce, S. C., Meyer, R. E., and Halmi, K. A. (2022). Affect Modulated Startle Response in Anorexia Nervosa, Restricting Type: Implications for Theory and Practice. *Cureus*. doi: 10.7759/cureus.27304

Martínez-García, C., Parra-Martínez, C., Parra, Á. T., Martínez-García, T. E., and Alameda-Bailén, J.-R. (2020). Iowa Gambling Task and Distortion in Perception of Body Image Among Adolescent Women With Eating Disorders. *Front Psychol* 11. doi: 10.3389/fpsyg.2020.02223

McAdams, C. J., Harper, J. A., and Van Enkevort, E. (2018). Mentalization and the left inferior frontal gyrus and insula. *European Eating Disorders Review* 26, 265–271. doi: 10.1002/erv.2580

McAdams, C. J., Jeon-Slaughter, H., Evans, S., Lohrenz, T., Montague, P. R., and Krawczyk, D. C. (2016). Neural differences in self-perception during illness and after weight-recovery in anorexia nervosa. *Soc Cogn Affect Neurosci* 11, 1823–1831. doi: 10.1093/scan/nsw092

McAdams, C. J., and Krawczyk, D. C. (2014). Who am I? How do I look? Neural differences in self-identity in anorexia nervosa. *Soc Cogn Affect Neurosci* 9, 12–21. doi: 10.1093/scan/nss093

Melles, H., and Jansen, A. (2024). Anxiety matters: a pilot lab study into food, weight, and virtual body exposure in anorexia nervosa. *J Eat Disord* 12, 141. doi: 10.1186/s40337-024-01094-w

Mendoza, C. J., Palka, J. M., Pelfrey, S. E., Hunt, B. J., Krawczyk, D. C., and McAdams, C. J. (2022). Neural processes related to negative self‐concept in adult and adolescent anorexia nervosa. *European Eating Disorders Review* 30, 23–35. doi: 10.1002/erv.2867

Meneguzzo, P., Dal Brun, D., Collantoni, E., Meregalli, V., Todisco, P., Favaro, A., et al. (2023). Linguistic embodiment in typical and atypical anorexia nervosa: Evidence from an image‐word matching task. *European Eating Disorders Review* 31, 837–849. doi: 10.1002/erv.3008

Meregalli, V., Giovannini, S., Trevisan, A., Romanelli, M., Ugur, S., Tenconi, E., et al. (2025). Eyes on the body: Assessing attentional bias toward body-related stimuli in Anorexia Nervosa. *J Psychiatr Res* 182, 506–512. doi: 10.1016/j.jpsychires.2025.01.043

Meregalli, V., Tenconi, E., Madan, C. R., Somà, E., Meneguzzo, P., Ceccato, E., et al. (2023). Beyond body image: what body schema and motor imagery can tell us about the way patients with anorexia nervosa experience their body. *Psychiatry Clin Neurosci* 77, 94–101. doi: 10.1111/pcn.13501

Mergen, J., Keizer, A., Koelkebeck, K., van den Heuvel, M. R. C., and Wagner, H. (2018). Women with Anorexia Nervosa do not show altered tactile localization compared to healthy controls. *Psychiatry Res* 267, 446–454. doi: 10.1016/j.psychres.2018.06.007

Metral, M., Guardia, D., Bauwens, I., Guerraz, M., Lafargue, G., Cottencin, O., et al. (2014). Painfully thin but locked inside a fatter body: abnormalities in both anticipation and execution of action in anorexia nervosa. *BMC Res Notes* 7, 707. doi: 10.1186/1756-0500-7-707

Miyake, Y., Okamoto, Y., Onoda, K., Shirao, N., Okamoto, Y., Otagaki, Y., et al. (2010). Neural processing of negative word stimuli concerning body image in patients with eating disorders: An fMRI study. *Neuroimage* 50, 1333–1339. doi: 10.1016/j.neuroimage.2009.12.095

Mohr, H. M., Zimmermann, J., Röder, C., Lenz, C., Overbeck, G., and Grabhorn, R. (2010). Separating two components of body image in anorexia nervosa using fMRI. *Psychol Med* 40, 1519–1529. doi: 10.1017/S0033291709991826

Mölbert, S. C., Thaler, A., Mohler, B. J., Streuber, S., Romero, J., Black, M. J., et al. (2018). Assessing body image in anorexia nervosa using biometric self-avatars in virtual reality: Attitudinal components rather than visual body size estimation are distorted. *Psychol Med* 48, 642–653. doi: 10.1017/S0033291717002008

Moody, T. D., Morfini, F., Cheng, G., Sheen, C. L., Kerr, W. T., Strober, M., et al. (2021). Brain activation and connectivity in anorexia nervosa and body dysmorphic disorder when viewing bodies: relationships to clinical symptoms and perception of appearance. *Brain Imaging Behav* 15, 1235–1252. doi: 10.1007/s11682-020-00323-5

Moody, T. D., Shen, V. W., Hutcheson, N. L., Henretty, J. R., Sheen, C. L., Strober, M., et al. (2017). Appearance evaluation of others’ faces and bodies in anorexia nervosa and body dysmorphic disorder. *International Journal of Eating Disorders* 50, 127–138. doi: 10.1002/eat.22604

Nandrino, J., Ducro, C., Iachini, T., and Coello, Y. (2017). Perception of Peripersonal and Interpersonal Space in Patients with Restrictive‐type Anorexia. *European Eating Disorders Review* 25, 179–187. doi: 10.1002/erv.2506

Nico, D., Daprati, E., Nighoghossian, N., Carrier, E., Duhamel, J.-R., and Sirigu, A. (2010). The role of the right parietal lobe in anorexia nervosa. *Psychol Med* 40, 1531–1539. doi: 10.1017/S0033291709991851

O’Hara, C. B., Keyes, A., Renwick, B., Giel, K. E., Campbell, I. C., and Schmidt, U. (2016). Evidence that Illness-Compatible Cues Are Rewarding in Women Recovered from Anorexia Nervosa: A Study of the Effects of Dopamine Depletion on Eye-Blink Startle Responses. *PLoS One* 11, e0165104. doi: 10.1371/journal.pone.0165104

Okamoto, Y., Miyake, Y., Shirao, N., Okamoto, Y., and Yamawaki, S. (2010). [Cognitive function in eating disorders and therapeutic approach]. *Seishin Shinkeigaku Zasshi* 112, 741–9.

Paquet, A., Girard, M., Passerieux, C., Boule, M.-C., Lacroix, A., Sazerat, P., et al. (2024). The body interior in anorexia nervosa: from interoception to conceptual representation of body interior. *Front Psychol* 15. doi: 10.3389/fpsyg.2024.1389463

Phillipou, A., Abel, L. A., Castle, D. J., Hughes, M. E., Gurvich, C., Nibbs, R. G., et al. (2015). Self perception and facial emotion perception of others in anorexia nervosa. *Front Psychol* 6. doi: 10.3389/fpsyg.2015.01181

Phillipou, A., Abel, L. A., Gurvich, C., Castle, D. J., and Rossell, S. L. (2020). Eye movements in anorexia nervosa: State or trait markers? *International Journal of Eating Disorders* 53, 1678–1684. doi: 10.1002/eat.23345

Phillipou, A., Rossell, S. L., Gurvich, C., Castle, D. J., Troje, N. F., and Abel, L. A. (2016a). Body Image in Anorexia Nervosa: Body Size Estimation Utilising a Biological Motion Task and Eyetracking. *European Eating Disorders Review* 24, 131–138. doi: 10.1002/erv.2423

Phillipou, A., Rossell, S. L., Gurvich, C., Hughes, M. E., Castle, D. J., Nibbs, R. G., et al. (2016b). Saccadic Eye Movements in Anorexia Nervosa. *PLoS One* 11, e0152338. doi: 10.1371/journal.pone.0152338

Pinhas, L., Fok, K.-H., Chen, A., Lam, E., Schachter, R., Eizenman, O., et al. (2014). Attentional biases to body shape images in adolescents with anorexia nervosa: An exploratory eye-tracking study. *Psychiatry Res* 220, 519–526. doi: 10.1016/j.psychres.2014.08.006

Pollatos, O., Herbert, B. M., Berberich, G., Zaudig, M., Krauseneck, T., and Tsakiris, M. (2016). Atypical Self-Focus Effect on Interoceptive Accuracy in Anorexia Nervosa. *Front Hum Neurosci* 10. doi: 10.3389/fnhum.2016.00484

Pona, A. A., Jones, A. C., Masterson, T. L., and Ben-Porath, D. D. (2019). Biases in attention and memory for body shape images in eating disorders. *Eating and Weight Disorders - Studies on Anorexia, Bulimia and Obesity* 24, 1165–1171. doi: 10.1007/s40519-017-0472-z

Porras-Garcia, B., Ferrer-Garcia, M., Serrano-Troncoso, E., Carulla-Roig, M., Soto-Usera, P., Miquel-Nabau, H., et al. (2020). Validity of Virtual Reality Body Exposure to Elicit Fear of Gaining Weight, Body Anxiety and Body-Related Attentional Bias in Patients with Anorexia Nervosa. *J Clin Med* 9, 3210. doi: 10.3390/jcm9103210

Provenzano, L., Ciccarone, S., Porciello, G., Petrucci, M., Cozzani, B., Cotugno, A., et al. (2024). Embodiment of underweight and normal-weight avatars affects bodily self-representations in anorexia nervosa. *Heliyon* 10, e32834. doi: 10.1016/j.heliyon.2024.e32834

Provenzano, L., Porciello, G., Ciccarone, S., Lenggenhager, B., Tieri, G., Marucci, M., et al. (2019). Characterizing Body Image Distortion and Bodily Self-Plasticity in Anorexia Nervosa via Visuo-Tactile Stimulation in Virtual Reality. *J Clin Med* 9, 98. doi: 10.3390/jcm9010098

Pruis, T. A., Keel, P. K., and Janowsky, J. S. (2012). Recovery from anorexia nervosa includes neural compensation for negative body image. *International Journal of Eating Disorders* 45, 919–931. doi: 10.1002/eat.22034

Radix, A. K., Sfärlea, A., Rinck, M., Becker, E. S., Platt, B., Schulte‐Körne, G., et al. (2023). Watch out! A path from anxiety to anorexia nervosa through biased attention? *European Eating Disorders Review* 31, 670–684. doi: 10.1002/erv.2994

Richard, A., Meule, A., Georgii, C., Voderholzer, U., Cuntz, U., Wilhelm, F. H., et al. (2019). Associations between interoceptive sensitivity, intuitive eating, and body mass index in patients with anorexia nervosa and normal‐weight controls. *European Eating Disorders Review* 27, 571–577. doi: 10.1002/erv.2676

Risso, G., Martoni, R. M., Erzegovesi, S., Bellodi, L., and Baud-Bovy, G. (2020). Visuo-tactile shape perception in women with Anorexia Nervosa and healthy women with and without body concerns. *Neuropsychologia* 149, 107635. doi: 10.1016/j.neuropsychologia.2020.107635

Rodriguez-Cano, T., Beato-Fernandez, L., Garcia-Vilches, I., Garcia-Vicente, A., Poblete-Garcia, V., and Soriano-Castrejon, A. (2009). Regional cerebral blood flow patterns of change following the own body image exposure in eating disorders: A longitudinal study. *European Psychiatry* 24, 275–281. doi: 10.1016/j.eurpsy.2008.11.004

Romero Frausto, H., Rahder, I., Dalhoff, A. W., Roesmann, K., Romer, G., Junghöfer, M., et al. (2024). Visual body size estimation in adolescent anorexia nervosa: Behavioural and neurophysiological data suggest intact visual perception and biased emotional attention. *Transl Psychiatry* 14, 442. doi: 10.1038/s41398-024-03144-y

Salvato, G., Sellitto, M., Crottini, F., Tarlarini, P., Tajani, M., Basilico, S., et al. (2024). Extreme weight conditions impact on the relationship between risky decision-making and interoception. *Cortex* 179, 126–142. doi: 10.1016/j.cortex.2024.07.009

Saramandi, A., Crucianelli, L., Koukoutsakis, A., Nisticò, V., Mavromara, L., Goeta, D., et al. (2024). Updating Prospective Self-Efficacy Beliefs About Cardiac Interoception in Anorexia Nervosa: An Experimental and Computational Study. *Computational Psychiatry* 8. doi: 10.5334/cpsy.109

Scarpina, F., Bastoni, I., Villa, V., Mendolicchio, L., Castelnuovo, G., Mauro, A., et al. (2022). Self-perception in anorexia nervosa: When the body becomes an object. *Neuropsychologia* 166, 108158. doi: 10.1016/j.neuropsychologia.2022.108158

Scarpina, F., Vaioli, G., Brusa, F., Bastoni, I., Villa, V., Mendolicchio, L., et al. (2024). Disgust drivers do not impact on the altered body in action representation in anorexia nervosa. *Quarterly Journal of Experimental Psychology*. doi: 10.1177/17470218241298668

Schecklmann, M., Pfannstiel, C., Fallgatter, A. J., Warnke, A., Gerlach, M., and Romanos, M. (2012). Olfaction in child and adolescent anorexia nervosa. *J Neural Transm* 119, 721–728. doi: 10.1007/s00702-011-0752-0

Sfärlea, A., Radix, A. K., Schulte-Körne, G., Legenbauer, T., and Platt, B. (2023). Attention Biases for Eating Disorder-Related Stimuli Versus Social Stimuli in Adolescents with Anorexia Nervosa – An Eye-Tracking Study. *Res Child Adolesc Psychopathol* 51, 541–555. doi: 10.1007/s10802-022-00993-3

Smith, A. R., Forrest, L. N., Velkoff, E. A., Ribeiro, J. D., and Franklin, J. (2018). Implicit attitudes toward eating stimuli differentiate eating disorder and non‐eating disorder groups and predict eating disorder behaviors. *International Journal of Eating Disorders* 51, 343–351. doi: 10.1002/eat.22843

Smith, A. R., Joiner, T. E., and Dodd, D. R. (2014). Examining implicit attitudes toward emaciation and thinness in anorexia nervosa. *International Journal of Eating Disorders* 47, 138–147. doi: 10.1002/eat.22210

Spitoni, G. F., Serino, A., Cotugno, A., Mancini, F., Antonucci, G., and Pizzamiglio, L. (2015). The two dimensions of the body representation in women suffering from Anorexia Nervosa. *Psychiatry Res* 230, 181–188. doi: 10.1016/j.psychres.2015.08.036

Spring, V. L., and Bulik, C. M. (2014). Implicit and explicit affect toward food and weight stimuli in anorexia nervosa. *Eat Behav* 15, 91–94. doi: 10.1016/j.eatbeh.2013.10.017

Stein, D., Gross-Isseroff, R., Besserglick, R., Ziv, A., Mayer, G., Yaroslavsky, A., et al. (2012). Olfactory function and alternation learning in eating disorders. *European Neuropsychopharmacology* 22, 615–624. doi: 10.1016/j.euroneuro.2011.12.006

Strigo, I. A., Matthews, S. C., Simmons, A. N., Oberndorfer, T., Klabunde, M., Reinhardt, L. E., et al. (2013). Altered insula activation during pain anticipation in individuals recovered from anorexia nervosa: Evidence of interoceptive dysregulation. *International Journal of Eating Disorders* 46, 23–33. doi: 10.1002/eat.22045

Susta, M., Bizik, G., Yamamotova, A., Petranek, S., Kadochova, M., and Papezova, H. (2022). The sight of one’s own body: Could qEEG help predict the treatment response in anorexia nervosa? *Front Psychol* 13. doi: 10.3389/fpsyg.2022.958501

Svaldi, J., Bender, C., Caffier, D., Ivanova, V., Mies, N., Fleischhaker, C., et al. (2016). Negative Mood Increases Selective Attention to Negatively Valenced Body Parts in Female Adolescents with Anorexia Nervosa. *PLoS One* 11, e0154462. doi: 10.1371/journal.pone.0154462

Sweitzer, M. M., Watson, K. K., Erwin, S. R., Winecoff, A. A., Datta, N., Huettel, S., et al. (2018). Neurobiology of social reward valuation in adults with a history of anorexia nervosa. *PLoS One* 13, e0205085. doi: 10.1371/journal.pone.0205085

Tagini, S., Bastoni, I., Villa, V., Mendolicchio, L., Castelnuovo, G., Mauro, A., et al. (2023). Affective touch in anorexia nervosa: Exploring the role of social anhedonia and lifespan experiences. *J Affect Disord* 324, 607–615. doi: 10.1016/j.jad.2022.12.137

Tajadura-Jiménez, A., Crucianelli, L., Zheng, R., Cheng, C., Ley-Flores, J., Borda-Más, M., et al. (2022). Body weight distortions in an auditory-driven body illusion in subclinical and clinical eating disorders. *Sci Rep* 12, 20031. doi: 10.1038/s41598-022-24452-7

Terhoeven, V., Nikendei, C., Faschingbauer, S., Huber, J., Young, K. D., Bendszus, M., et al. (2023). Neurophysiological correlates of disorder-related autobiographical memory in anorexia nervosa. *Psychol Med* 53, 844–854. doi: 10.1017/S003329172100221X

Tonacci, A., Calderoni, S., Billeci, L., Maestro, S., Fantozzi, P., Ciuccoli, F., et al. (2019). Autistic traits impact on olfactory processing in adolescent girls with Anorexia Nervosa restricting type. *Psychiatry Res* 274, 20–26. doi: 10.1016/j.psychres.2019.02.026

Tuschen-Caffier, B., Bender, C., Caffier, D., Klenner, K., Braks, K., and Svaldi, J. (2015). Selective Visual Attention during Mirror Exposure in Anorexia and Bulimia Nervosa. *PLoS One* 10, e0145886. doi: 10.1371/journal.pone.0145886

Urgesi, C., Fornasari, L., Canalaz, F., Perini, L., Cremaschi, S., Faleschini, L., et al. (2014). Impaired configural body processing in anorexia nervosa: Evidence from the body inversion effect. *British Journal of Psychology* 105, 486–508. doi: 10.1111/bjop.12057

Urgesi, C., Fornasari, L., Perini, L., Canalaz, F., Cremaschi, S., Faleschini, L., et al. (2012). Visual body perception in anorexia nervosa. *International Journal of Eating Disorders* 45, 501–511. doi: 10.1002/eat.20982

Via, E., Goldberg, X., Sánchez, I., Forcano, L., Harrison, B. J., Davey, C. G., et al. (2018). Self and other body perception in anorexia nervosa: The role of posterior DMN nodes. *The World Journal of Biological Psychiatry* 19, 210–224. doi: 10.1080/15622975.2016.1249951

Vocks, S., Busch, M., Grönemeyer, D., Schulte, D., Herpertz, S., and Suchan, B. (2010). Neural correlates of viewing photographs of one’s own body and another woman’s body in anorexia and bulimia nervosa: an fMRI study. *Journal of Psychiatry and Neuroscience* 35, 163–176. doi: 10.1503/jpn.090048

Voges, M. M., Giabbiconi, C., Schöne, B., Braks, K., Huber, T. J., Waldorf, M., et al. (2018). Double standards in body evaluation? How identifying with a body stimulus influences ratings in women with anorexia nervosa and bulimia nervosa. *International Journal of Eating Disorders* 51, 1223–1232. doi: 10.1002/eat.22967

von Wietersheim, J., Kunzl, F., Hoffmann, H., Glaub, J., Rottler, E., and Traue, H. C. (2012). Selective Attention of Patients With Anorexia Nervosa While Looking at Pictures of Their Own Body and the Bodies of Others. *Psychosom Med* 74, 107–113. doi: 10.1097/PSY.0b013e31823ba787

Waldman, A., Loomes, R., Mountford, V. A., and Tchanturia, K. (2013). Attitudinal and perceptual factors in body image distortion: an exploratory study in patients with anorexia nervosa. *J Eat Disord* 1, 17. doi: 10.1186/2050-2974-1-17

Watson, K. K. (2010). Altered social reward and attention in anorexia nervosa. *Front Psychol* 1. doi: 10.3389/fpsyg.2010.00036

Xu, J., Harper, J. A., Van Enkevort, E. A., Latimer, K., Kelley, U., and McAdams, C. J. (2017). Neural activations are related to body-shape, anxiety, and outcomes in adolescent anorexia nervosa. *J Psychiatr Res* 87, 1–7. doi: 10.1016/j.jpsychires.2016.12.005

Yokokura, M., Terada, T., Bunai, T., Nakaizumi, K., Kato, Y., Yoshikawa, E., et al. (2019). Alterations in serotonin transporter and body image-related cognition in anorexia nervosa. *Neuroimage Clin* 23, 101928. doi: 10.1016/j.nicl.2019.101928
